# A study on differences in lung function development and longitudinal patterns between preterm infants of different gestational ages and term infants

**DOI:** 10.3389/fped.2026.1763663

**Published:** 2026-06-23

**Authors:** Rongjie Chen, Xiao Liu, Zhe Xu

**Affiliations:** 1 Department of Pediatrics, Guangyuan Central Hospital Affiliated to North Sichuan Medical College, Nanchong, Sichuan, China; 2 Department of Pediatrics, Guangyuan Central Hospital, Guangyuan, Sichuan, China

**Keywords:** compensatory recuperation, developmental discrepancies, full-term neonates, gestational duration, longitudinal alterations, preterm neonates, pulmonary functionality

## Abstract

**Objective:**

To address gaps in small airway function follow-up and grouping criteria for extremely preterm infants, this study adopted dual grouping to explore baseline differences and longitudinal pulmonary function patterns between preterm (by gestational age: < 32 weeks, 32–33 + 6 weeks, 34–36 + 6 weeks) and term (≥ 37 weeks) infants. It aimed to clarify impacts of gestational age, birth weight, and other factors, providing evidence for individualized clinical follow-up and intervention.

**Methods:**

From 2020 to 2024, 113 term-equivalent infants from Guangyuan Central Hospital were enrolled. Lung function parameters (VT, TPEF/TE, TEF50, etc.) at corrected 40 weeks (0 months), 3 and 6 months, plus perinatal data were collected. Inter-group differences were analyzed via ANOVA/Kruskal–Wallis tests, baseline factors via linear regression, longitudinal change rates via linear mixed models (LMM), and intra-group recovery via repeated-measures ANOVA.

**Results:**

At baseline, lower gestational age correlated with reduced VT, TPEF/TE, VPEF/VE, and elevated RR (*P* < 0.05); the < 32 weeks group had significantly lower VT (20.4 + 4.6 mL) vs. ≥ 37 weeks (24.6 + 3.1 mL, *P* < 0.001). At 3 months, < 32 weeks infants had higher VT (51.6 + 8.3 mL) than 34–36 + 6 weeks (39.5 + 9.2 mL, *P* = 0.001). At 6 months, only TEF50 in < 32 weeks group (84.2 + 17.6 mL/s) remained lower vs. ≥ 37 weeks (75.1 + 19.7 mL/s, *P* < 0.05). LMM showed each additional week of gestational age increased VT by 0.15 mL/month (*P* = 0.017)); < 32 weeks group had faster VT growth (0.23 mL/month) than ≥ 37 weeks (0.09 mL/month, *P* < 0.05). Each 1 kg higher birth weight increased VT by 0.75 mL/month (*P* = 0.003); maternal gestational diabetes accelerated VT growth (*β* = 1.04 mL/month *P* = 0.025).

**Conclusion:**

Preterm infants exhibit gestational age-dependent compensatory lung function recovery, with faster rates in younger gestational ages. However, extremely preterm infants (< 32 weeks) infants (< 32 weeks) have delayed small airway function (TEF50) at 6 months. Gestational age, birth weight, and maternal gestational diabetes are key factors. Clinicians should implement individualized 3-monthly small airway function monitoring for < 32 weeks infants to reduce long-term respiratory disease risk.

## 1 Introduction

Preterm infants exhibit significantly elevated risks of long-term respiratory diseases (e.g., chronic obstructive pulmonary disease and recurrent wheezing) in comparison to term infants, attributable to incomplete alveolar differentiation and insufficient pulmonary surfactant synthesis (1–3). Abnormal trajectories of pulmonary function development serve as the core contributing factors. Tidal breathing pulmonary function testing has emerged as the preferred approach for neonatal pulmonary function assessment due to its non-invasive characteristics and operational simplicity (4, 5).

Tidal volume (VT) reflects basal alveolar ventilation capacity; reduced VT indicates immature alveolar development (6). The ratio of time to peak expiratory flow to total expiratory time (TPEF/TE) is a sensitive marker of small airway patency; a lower value reflects increased early airway resistance (7). Expiratory flow at 50% tidal volume (TEF50) directly reflects small airway function and is closely associated with long-term wheezing and asthma risk (8, 9). Respiratory rate (RR) indirectly reflects ventilatory efficiency; higher RR suggests compensatory tachypnea due to poor lung compliance (10).

Nevertheless, existing studies are confronted with three major limitations:

(a) There is a dearth of longitudinal follow-up regarding small airway function (e.g., TEF50) in extremely preterm infants (< 32 weeks), which impedes the identification of critical milestones for catch-up development (11, 12).
(b) Single-group classification schemes are predominantly employed, lacking validation of the stability of conclusions (13, 14).
(c) There is an inadequate analysis of the interactions between perinatal factors (e.g., birth weight, maternal gestational diabetes) and gestational age (15).

Consequently, this study implements a dual design of “refined four-group comparison + expanded three-group validation” with a 6-month longitudinal follow-up. It centers on the alterations in small airway function in extremely preterm infants to elucidate the influence of gestational age and perinatal factors, thus providing evidence for the formulation of precise clinical follow-up and intervention strategies.

## 2 Materials and methods

### 2.1 Study population

A total of 116 newborns admitted to the Neonatal Department of our hospital between January 2020 and December 2024 were selected. Three cases with missing baseline pulmonary function data were excluded, leaving 113 eligible participants.

**Inclusion criteria:**

(a) Term-equivalent infants (birth weight within the P10–90 percentile for gestational age);
(b) Survival and follow-up until 6 months of age.

**Exclusion criteria:**

Congenital heart disease, airway structural abnormalities, inherited metabolic disorders, or other conditions severely impairing lung function; or refusal of informed consent by the family.

**Grouping scheme:**

(a) Four subgroups (refining preterm subgroups): < 32 weeks (extremely preterm), 32–33 + 6 weeks (late preterm), 34–36 + 6 weeks (near-term), ≥ 37 weeks (term);
(b) Three-group scheme (to enhance statistical power): < 34 weeks (extremely preterm + late preterm), 34–36 + 6 weeks, 37–42 weeks, with term infants as the control group.

### 2.2 Data collection and preprocessing

Basic data included gestational age at birth, birth weight, maternal age, and perinatal complications. Lung function parameters (VT, TPEF/TE, TEF50, etc.) were measured using the Jaeger MasterScreen Baby spirometer in a dedicated laboratory maintained at 22–25 °C and 50%–60% humidity. Five valid curves (coeficient of variation < 10% were collected per session, and the mean value was recorded.

**Measurement timepoints:** 0 months (corrected gestational age 40 weeks), 3 months, 6 months (all calculated based on chronological age).

**Data preprocessing:** Core variable missing values were labeled as NA; covariates with a missing rate of < 10% required no imputation. Outliers identified using “mean + 3 standard deviations” were included after clinical verification. New variables were generated: gestational age groups, respiratory comorbidities, etc.

### 2.3 Statistical analysis

R 4.3.0 software was used; the significance level *α* = 0.05.

(a) Descriptive statistics: Quantitative data were presented as “x¯ ± s”; categorical data as “frequency (%)”.
(b) Intergroup comparisons: ANOVA/Kruskal–Wallis test.
(c) Regression analysis: Linear regression (adjusted for gestational age and birth weight) was used to analyze baseline effects; linear mixed models (LMM) incorporated individual-level random intercepts to analyze longitudinal change rates (effect size represented by the “variable ×  time” interaction term), adjusted for covariates including birth weight and maternal age.
(d) Repeated measures analysis: Mauchly's test for sphericity; if the sphericity assumption was not satisfied, the Greenhouse-Geisser correction was applied; Bonferroni multiple comparisons were used for within-group comparisons.e. Visualization: Line charts (with standard error bars) and box plots were used to present results.

## 3 Results

### 3.1 Comparison of basic characteristics and baseline pulmonary function among neonates in different gestational age quartiles

Among 113 newborns, the < 32-week group had a mean gestational age at birth of (30.5 ± 1.2) weeks and a birth weight of (1.6 ± 0.3) kg; the ≥ 37-week group had a mean gestational age at birth of (39.0 ± 1.1) weeks and a birth weight of (3.3 ± 0.5) kg. With increasing gestational age, birth weight and birth length showed an increasing trend (*P* < 0.001). The incidence of RDS (63.2%) and the use of noninvasive ventilation (78.9%) were significantly higher in the < 32-week group than in the ≥ 37-week group (25.0%, 25.0%) (*P* < 0.05) (Table 1).

**Table 1:** Comparison of basic characteristics and baseline pulmonary function among neonates in four gestational age groups (*N* = 113).

Characteristic	Overall	<32W(*n* = 19)	32–33 + 6 W (*n* = 21)	34–36 + 6 W (*n* = 37)	≥37W (*n* = 36)	*p*-value
Gestational age at birth (W)	35.3 ± 3.2	30.5 ± 1.2	32.8 ± 0.5	35.2 ± 0.8	39.0 ± 1.1	<0.001
Birth weight (kg)	2.4 ± 0.8	1.6 ± 0.3	1.8 ± 0.3	2.2 ± 0.4	3.3 ± 0.5	<0.001
Birth height (cm)	45.8 ± 3.9	41.2 ± 2.1	42.5 ± 1.8	44.8 ± 2.5	49.8 ± 1.5	<0.001
Mother's age (Y)	28.8 ± 4.2	29.5 ± 4.8	28.2 ± 3.9	28.6 ± 4.3	28.0 ± 2.6	0.892
Apgar Score-1min	8.5 ± 1.4	7.8 ± 1.8	8.2 ± 1.5	8.6 ± 1.3	8.9 ± 1.4	0.215
Apgar Score-5min	9.4 ± 0.9	8.9 ± 1.2	9.2 ± 1.0	9.5 ± 0.8	9.8 ± 0.7	0.068
Premature Rupture of Membranes (%)	27 (23.9%)	5 (26.3%)	6 (28.6%)	10 (27.0%)	6 (16.7%)	0.645
Gestational diabetes (%)	20 (17.7%)	5 (26.3%)	4 (19.0%)	7 (18.9%)	4 (11.1%)	0.538
Fetal Distress (%)	17 (15.0%)	4 (21.1%)	3 (14.3%)	6 (16.2%)	4 (11.1%)	0.763
Neonatal Resuscitation (Positive Pressure Ventilation) (%)	30 (26.5%)	8 (42.1%)	7 (33.3%)	9 (24.3%)	6 (16.7%)	0.187
Use of noninvasive ventilation (%)	52 (46.0%)	15 (78.9%)	12 (57.1%)	16 (43.2%)	9 (25.0%)	<0.001
Merge RDS (%)	45 (39.8%)	12 (63.2%)	9 (42.9%)	15 (40.5%)	9 (25.0%)	0.038
VT, mL	21.9 ± 4.5	20.4 ± 4.6	19.0 ± 3.5	21.8 ± 4.7	24.6 ± 3.1	<0.001
VT/kg, mL/kg	7.2 ± 1.2	7.2 ± 0.9	6.9 ± 1.0	7.3 ± 1.4	7.3 ± 1.1	0.395
TPEF/TE (%)	27.8 ± 9.8	18.9 ± 5.2	27.6 ± 8.1	27.4 ± 6.7	33.4 ± 11.9	<0.001
VPEF/VE (%)	29.8 ± 8.5	22.9 ± 4.3	29.8 ± 6.6	29.1 ± 5.5	34.6 ± 10.9	<0.001
TEF75, mL/s	58.4 ± 13.0	59.1 ± 11.4	57.7 ± 14.4	56.2 ± 14.7	60.9 ± 10.8	0.535
TEF50, mL/s	54.0 ± 11.6	49.4 ± 7.4	56.1 ± 11.9	52.0 ± 13.2	57.5 ± 10.5	0.029
RR, (breaths per minute)	57.8 ± 11.4	58.0 ± 8.9	67.4 ± 12.1	56.4 ± 11.9	53.8 ± 8.4	0.001
Ti, s	0.5 ± 0.1	0.5 ± 0.1	0.4 ± 0.1	0.5 ± 0.1	0.5 ± 0.1	0.019
Te, s	0.6 ± 0.1	0.6 ± 0.1	0.5 ± 0.1	0.6 ± 0.2	0.6 ± 0.1	0.023
Ti/Te	0.8 ± 0.1	0.7 ± 0.1	0.8 ± 0.1	0.8 ± 0.1	0.8 ± 0.1	0.016

W, week; Y, year.

Baseline pulmonary function (corrected to 40 weeks' gestational age): Lower gestational age was associated with lower VT, TPEF/TE, and VPEF/VE, and higher RR (*P* < 0.05)a. The VT (20.4 ± 4.6 mL) in the < 32-week group was significantly lower than that in the 34–36 + 6-week group (21.8 + 4.7 mL) and the ≥ 37-week group (24.6 + 3.1 mL) (*P* < 0.001)b. RR (67.4 ± 12.1 breaths/min) in the 32–33 + 6-week group was significantly higher than in the other three groups (*P* = 0.001)c. The TPEF/TE (18.9 ± 5.2%) and VPEF/VE (22.9 ± 4.3%) in the < 32-week group were significantly lower than those in the ≥ 37-week group (33.4 ± 11.9%, 34.6 ± 10.9%) (*P* < 0.001).

### 3.2 Comparison of basic characteristics and baseline pulmonary function among neonates in three gestational age groups

The < 34-week group had a mean gestational age at birth of (31.6 ± 1.5) weeks and a mean birth weight of (1.7 ± 0.3) kg. The 37–42-week group had a mean gestational age at birth of (39.3 ± 1.2) weeks and a mean birth weight of (3.3 ± 0.5) kg. Apgar scores at 5 min showed an increasing trend with gestational age (*P* = 0.029). The non-invasive ventilation utilization rate in the < 34-week group (67.5%) was significantly higher than that in the 37–42-week group (25.0%) (*P* < 0.001) (Table 2).

**Table 2:** Comparison of basic characteristics and baseline pulmonary function Among neonates in three gestational Age groups (*N* = 113).

Characteristic	Overall	< 34 W (*n* = 40)	34–36 + 6 W (*n* = 37)	37–42 W (*n* = 36)	*p*-value
Gestational age at birth (W)	35.3 ± 3.2	31.6 ± 1.5	35.2 ± 0.8	39.3 ± 1.2	< 0.001
Birth weight (kg)	2.4 ± 0.8	1.7 ± 0.3	2.2 ± 0.4	3.3 ± 0.5	< 0.001
Birth height (cm)	45.8 ± 3.9	41.8 ± 2.0	44.8 ± 2.5	49.9 ± 1.4	< 0.001
Mother's age (Y)	28.8 ± 4.2	28.9 ± 4.4	28.6 ± 4.3	28.0 ± 2.6	0.867
Apgar Score-1min	8.5 ± 1.4	8.0 ± 1.7	8.6 ± 1.3	8.9 ± 1.4	0.128
Apgar Score-5min	9.4 ± 0.9	9.0 ± 1.1	9.5 ± 0.8	9.8 ± 0.7	0.029
Premature Rupture of Membranes (%)	27 (23.9%)	11 (27.5%)	10 (27.0%)	6 (16.7%)	0.512
Gestational diabetes (%)	20 (17.7%)	9 (22.5%)	7 (18.9%)	4 (11.1%)	0.408
Fetal Distress (%)	17 (15.0%)	7 (17.5%)	6 (16.2%)	4 (11.1%)	0.715
Neonatal Resuscitation (Positive Pressure Ventilation) (%)	30 (26.5%)	15 (37.5%)	9 (24.3%)	6 (16.7%)	0.116
Use of noninvasive ventilation (%)	52 (46.0%)	27 (67.5%)	16 (43.2%)	9 (25.0%)	< 0.001
Merge RDS (%)	45 (39.8%)	21 (52.5%)	15 (40.5%)	9 (25.0%)	0.045
VT，mL	21.9 ± 4.5	19.7 ± 4.1	21.8 ± 4.7	24.6 ± 3.1	< 0.001
VT/kg，mL/kg	7.2 ± 1.2	7.0 ± 0.9	7.3 ± 1.4	7.3 ± 1.1	0.405
TPEF/TE (%)	27.8 ± 9.8	23.4 ± 8.0	27.4 ± 6.7	33.4 ± 11.9	< 0.001
VPEF/VE (%)	29.8 ± 8.5	26.4 ± 6.5	29.1 ± 5.5	34.6 ± 10.9	0.001
TEF75, mL/s	58.4 ± 13.0	58.4 ± 12.9	56.2 ± 14.7	60.9 ± 10.8	0.346
TEF50, mL/s	54.0 ± 11.6	52.8 ± 10.4	52.0 ± 13.2	57.5 ± 10.5	0.060
RR, (breaths per minute)	57.8 ± 11.4	62.8 ± 11.5	56.4 ± 11.9	53.8 ± 8.4	0.005
Ti，s	0.5 ± 0.1	0.4 ± 0.1	0.5 ± 0.1	0.5 ± 0.1	0.009
Te，s	0.6 ± 0.1	0.6 ± 0.1	0.6 ± 0.2	0.6 ± 0.1	0.641
Ti/Te	0.8 ± 0.1	0.8 ± 0.1	0.8 ± 0.1	0.8 ± 0.1	0.531

**Baseline pulmonary function:**

(a) The < 34-week group had significantly lower VT (19.7 ± 4.1 mL) and TPEF/TE (23.4 ± 8.0%) compared to the 37–42-week group (24.6 ± 3.1 mL, 33.4 ± 11.9%) (*P* < 0.001).
(b) RR (62.8 + 11.5 breaths/min) in the < 34-week group was significantly higher than that in the 34–36 + 6-week group (56.4 ± 11.9 breaths/min) and the 37–42-week group (53.8 ± 8.4 breaths/min) (*P* = 0.005).

### 3.3 Effects of gestational age groups on neonatal baseline pulmonary function

#### 3.3.1 Effects of gestational age quartiles (adjusted for birth gestational age and birth weight)

Compared with the ≥ 37 weeks group, the < 32 weeks group exhibited a reduction in VT of 8.79 mL (*P* = 0.024) and a decline in TPEF/TE of 21.60% (*P* = 0.015).In the 32–33 + 6 weeks group, VT decreased by 8.52 mL (*P* = 0.002), and in the 34–36 + 6 weeks group, it decreased by 3.96 mL (*P* = 0.031)In the < 32 weeks group, TEF50 decreased by 21.44 mL/s (*P* = 0.053), approaching statistical significance (Table 3).

**Table 3:** Linear regression analysis of the effects of gestational age groups on baseline pulmonary function indicators in newborns.

Outcome	Grouping Scheme	Control group	Control group	Crude model		Model 1	
				β (95%CI)	*p*-value	β (95%CI)	*p*-value
VT, mL	Quadrant	≥ 37 W	< 32 W	−4.16 (−6.44, −1.87)	< 0.001	−8.79 (−16.38, −1.20)	0.024
			32–33 + 6 W	−5.58 (−7.83, −3.33)	< 0.001	−8.52 (−13.72, −3.31)	0.002
			34–36 + 6 W	−2.83 (−4.72, −0.93)	0.004	−3.96 (−7.54, −0.37)	0.031
	Group of three	37–42 W	< 34 W	−4.89 (−6.76, −3.01)	< 0.001	−8.33 (−12.88, −3.77)	< 0.001
			34–36 + 6 W	−2.83 (−4.73, −0.93)	0.004	−3.79 (−6.68, −0.91)	0.011
VT/kg, mL/kg	Quadrant	≥ 37 W	< 32 W	−0.09 (−0.75, 0.57)	0.791	−1.78 (−3.98, 0.41)	0.111
			32–33 + 6 W	−0.44 (−1.09, 0.21)	0.185	−1.51 (−3.01, −0.00)	0.050
	Group of three	37–42 W	< 34 W	−0.27 (−0.81, 0.27)	0.329	−1.32 (−2.64, 0.00)	0.050
TPEF/TE, (%)	Quadrant	≥ 37W	< 32 W	−14.50 (−19.45, −9.55)	< 0.001	−21.60 (−38.90, −4.31)	0.015
RR, (breaths per minute)	Group of three	37–42 W	< 34 W	9.02 (4.00, 14.04)	< 0.001	17.22 (4.77, 29.68)	0.007
TEF50, mL/s	Quadrant	≥ 37W	< 32 W	−8.16 (−14.58, −1.74)	0.013	−21.44 (−43.20, 0.33)	0.053

Model 1 adjusted for gestational age at birth and birth weight; baseline lung function was the first measurement at 40 weeks' corrected gestational age.

#### 3.3.2 Effects of gestational age stratification (adjusted for birth gestational age and weight)

Compared with the 37–42 weeks group:The < 34 weeks group showed a decrease in VT of 8.33 mL (*P* < 0.001) and an increase in RR of 17.22 beats/min (*P* = 0.007).In the 34–36 + 6 weeks group, VT decreased by 3.79 mL (*P* = 0.011)In the < 34 weeks group, VT/kg decreased by 1.32 mL/kg (*P* = 0.050), approaching statistical significance.

### 3.4 Longitudinal patterns of pulmonary function changes in neonates across gestational age groups

Linear mixed models (LMM) revealed the following effect values representing the rate of change in pulmonary function over time (months): (Table 4)

(a) Total Cohort: For each additional week of gestational age, VT increased by 0.15 mL/month (*P* = 0.017), TPEF/TE decreased by 0.16%/month (*P* = 0.002), and VPEF/VE decreased by 0.12%/month (*P* = 0.002). This decline in TPEF/TE reflects physiological maturation of breathing patterns, not pathological airway deterioration.
(b) By gestational age group: The VT growth rate in the < 32-weeks group (0.23 mL/month, *P* = 0.009) was higher than that in the 32–33 + 6 weeks group (0.19 mL/month, *P* = 0.022), the 34–36 + 6 weeks group (0.14 mL/month, *P* = 0.038), and the ≥ 37 weeks group (0.09 mL/month, *P* = 0.085). The rate of TPEF/TE reduction in the ≥ 37 weeks group (−0.21%/month, *P* = 0.002) was greater than that in the < 32 weeks group (−0.11%/month, *P* = 0.092).
(c) Other factors: For every 1-kg increase in birth weight, VT increased by 0.75 mL/month (*P* = 0.003). VT growth accelerated in newborns with maternal gestational diabetes (*β* = 1.04 mL/month, *P* = 0.025), while RR decreased by 1.51 beats/min/month (*P* = 0.009)

**Table 4:** Linear mixed model analysis of the effects of perinatal factors on longitudinal changes in pulmonary function among neonates of different gestational age groups.

Outcome	Gestational Age Groups	Crude model		Model 1	
		β (95%CI)	*p*-value	β (95%CI)	*p*-value
VT, mL/m	Overall	0.15 (0.03, 0.28)	0.015	0.15 (0.03, 0.28)	0.017
	< 32 W	0.22 (0.05, 0.39)	0.012	0.23 (0.06, 0.40)	0.009
	32–33 + 6W	0.18 (0.02, 0.34)	0.028	0.19 (0.03, 0.35)	0.022
	34–36 + 6 W	0.14 (0.01, 0.27)	0.035	0.14 (0.01, 0.27)	0.038
	≥ 37 W	0.08 (−0.02, 0.18)	0.112	0.09 (−0.01, 0.19)	0.085
TPEF/TE, %/ m	Overall	−0.17 (−0.28, −0.07)	< 0.001	−0.16 (−0.27, −0.06)	0.002
	< 32 W	−0.12 (−0.25, 0.01)	0.068	−0.11 (−0.24, 0.02)	0.092
	32–33 + 6W	−0.18 (−0.30, −0.06)	0.004	−0.17 (−0.29, −0.05)	0.006
	34–36 + 6W	−0.19 (−0.31, −0.07)	0.002	−0.18 (−0.30, −0.06)	0.003
	≥ 37W	−0.22 (−0.35, −0.09)	0.001	−0.21 (−0.34, −0.08)	0.002
TEF50, mL/s/ m	Overall	0.14 (−0.08, 0.35)	0.212	0.15 (−0.08, 0.37)	0.194
	< 32 W	0.11 (−0.12, 0.34)	0.345	0.10 (−0.13, 0.33)	0.382
RR, (breaths per minute)	Overall	0.02 (−0.13, 0.18)	0.750	0.04 (−0.12, 0.20)	0.610

(m:month) The effect value represents the “variable  ×  time” interaction term; Model 1 adjusted for birth weight, maternal age, and gestational diabetes; only key pulmonary function indicators are shown.

### 3.5 Pulmonary function recovery in neonates of different gestational age groups

Repeated-measures ANOVA revealed significant differences in pulmonary function across different ages within the same gestational age group: (Table 5)

(a) VT: A significant increase was observed from 0 to 6 months across all gestational age groups (*P* < 0.001). The greatest increase was seen in the < 32-weeks group (43.0 ± 10.2 mL), while the smallest increase was observed in the ≥ 37-weeks group (35.2 ± 9.5 mL).
(b) RR: There was a significant decrease from 0 to 3 months across all gestational age groups (*P* < 0.001), with no significant change from 3 to 6 months (*P* > 0.05). The greatest decrease (34.1 ± 10.5 breaths/min) occurred in the 32–33 + 6-weeks group.
(c) TEF50: The < 32-weeks group showed a significant increase from 0 months (49.4 ± 7.4 mL/s) to 6 months (84.2 ± 17.6 mL/s) (*P* < 0.001), but remained lower than the ≥ 37-weeks group (75.1 ± 19.7 mL/s) at 6 months (*P* < 0.05).
(d) TPEF/TE: The TPEF/TE ratio decreased significantly from 0 months to 6 months in the 32–33 + 6-weeks, 34–36 + 6-weeks, and ≥ 37-weeks groups (*P* < 0.001), while no significant change was observed in the < 32-weeks group (*P* = 0.582).

**Table 5:** Comparison of pulmonary function recovery in neonates of different gestational Age groups at different ages (x ± s).

Pulmonary function indicators	Gestational Age Groups	0 months old	3 months old	6 months old	*F*-value/*P*-value (0 vs. 3 months)	*F*-value/*P*-value (3 vs. 6 months)	*F*-value/*P*-value (0 vs. 6 months)
VT, mL	< 32 W	20.4 ± 4.6	51.6 ± 8.3	63.4 ± 9.7	42.86/< 0.001	8.25/0.008	78.52/< 0.001
	32–33 + 6 W	19.0 ± 3.5	44.0 ± 9.8	56.2 ± 9.6	35.17/< 0.001	5.91/0.022	68.34/< 0.001
	34–36 + 6 W	21.8 ± 4.7	39.5 ± 9.2	64.0 ± 13.5	28.63/< 0.001	14.72/< 0.001	92.15/< 0.001
	≥ 37 W	24.6 ± 3.1	48.6 ± 9.6	59.8 ± 8.1	31.05/< 0.001	4.83/0.035	65.78/< 0.001
TPEF/TE, %	< 32 W	18.9 ± 5.2	19.5 ± 4.2	19.7 ± 5.2	0.18/0.675	0.04/0.841	0.31/0.582
	32–33 + 6 W	27.6 ± 8.1	18.6 ± 5.7	16.9 ± 4.6	10.25/0.004	0.87/0.360	22.43/< 0.001
	34–36 + 6 W	27.4 ± 6.7	21.1 ± 6.3	18.8 ± 5.0	8.92/0.005	2.98/0.095	24.67/< 0.001
	≥ 37 W	33.4 ± 11.9	19.8 ± 5.3	20.2 ± 3.8	23.56/< 0.001	0.07/0.792	22.14/< 0.001
TEF50, mL/s	< 32 W	49.4 ± 7.4	68.0 ± 18.0	84.2 ± 17.6	7.28/0.012	5.13/0.032	28.95/< 0.001
	32–33 + 6 W	56.1 ± 11.9	64.8 ± 13.7	70.3 ± 20.3	1.89/0.182	0.65/0.427	3.12/0.088
	34–36 + 6 W	52.0 ± 13.2	67.0 ± 10.4	83.9 ± 15.8	8.57/0.006	10.38/0.003	35.71/< 0.001
	≥ 37 W	57.5 ± 10.5	72.9 ± 15.3	75.1 ± 19.7	6.93/0.013	0.19/0.666	10.82/0.002
RR, (breaths per minute)	< 32 W	58.0 ± 8.9	33.1 ± 4.9	32.9 ± 6.6	38.74/< 0.001	0.01/0.921	40.15/< 0.001
	32–33 + 6 W	67.4 ± 12.1	38.1 ± 8.2	33.3 ± 6.0	45.29/< 0.001	3.28/0.081	78.56/< 0.001
	34–36 + 6 W	56.4 ± 11.9	40.7 ± 7.6	32.5 ± 6.2	26.31/< 0.001	12.45/< 0.001	68.93/< 0.001
	≥ 37W	53.8 ± 8.4	36.1 ± 6.9	31.7 ± 5.3	29.87/< 0.001	5.72/0.024	58.64/< 0.001

Repeated measures analysis of variance (ANOVA) was employed. The *F*-value represents the ANOVA statistic, while the *P*-value indicates the significance of differences between time points within groups.

### 2.6 Visualization of pulmonary development trajectories

**Figure 1:**
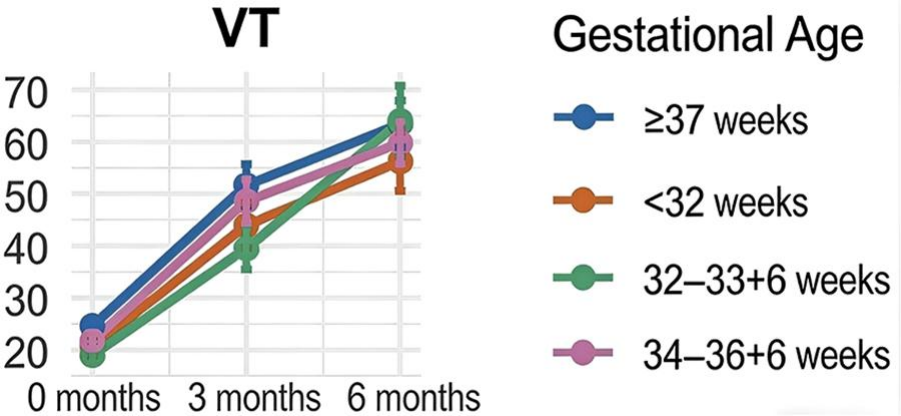
Trend line chart of tidal volume (VT, mL) in newborns of different gestational ages according to month of Age. Vertical axis: VT value, unit mL; Horizontal axis: Gestational age (0 months, 3 months, 6 months); Error bars: Standard deviation; At 6 months, the VT value in the < 32-week group (63.4 ± 9.7 mL) approached that of the ≥ 37-week group (59.8 ± 8.1 mL).

**Figure 2:**
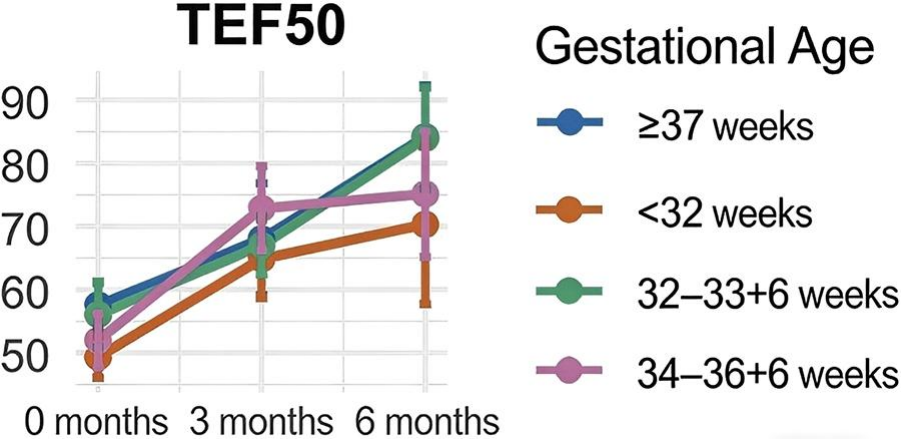
Trend line chart showing changes in expiratory flow rate at 50% tidal volume (TEF50, mL/s) with age in newborns of different gestational ages. (Vertical axis: TEF50 values, units mL/s; Horizontal axis: Gestational age; < 32 weeks group still exhibits lower TEF50 values at 6 months compared to ≥ 37 weeks group).

**Figure 3:**
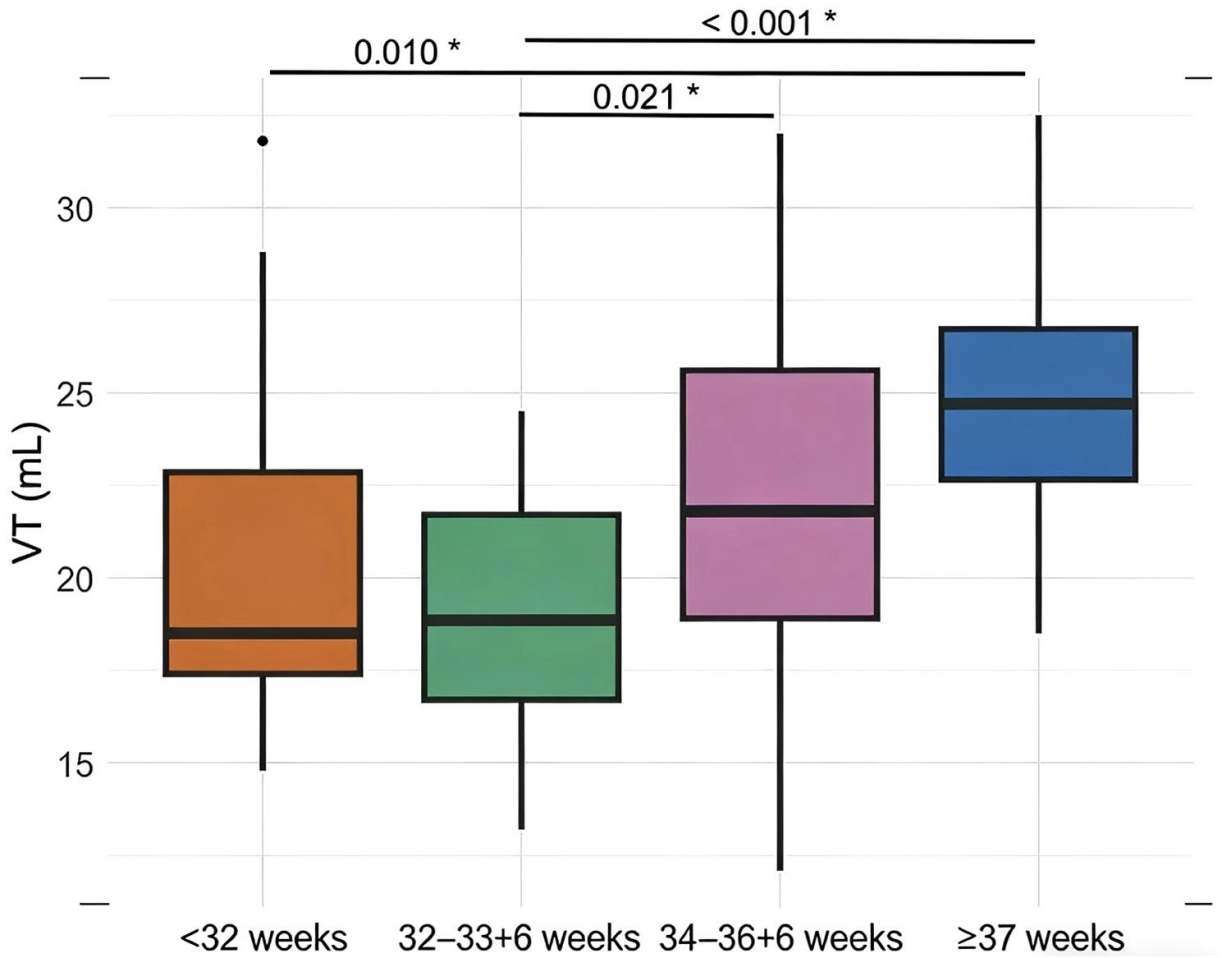
Box-and-whisker plot showing baseline tidal volume (VT, mL) distribution among newborns grouped by gestational age (corrected to 40 weeks). [Vertical axis: VT value, unit mL; Box plot: interquartile range (Q1-Q3); Horizontal line: median; Significant difference between < 32 weeks and ≥ 37 weeks groups (*P* < 0.001)].

**Figure 4:**
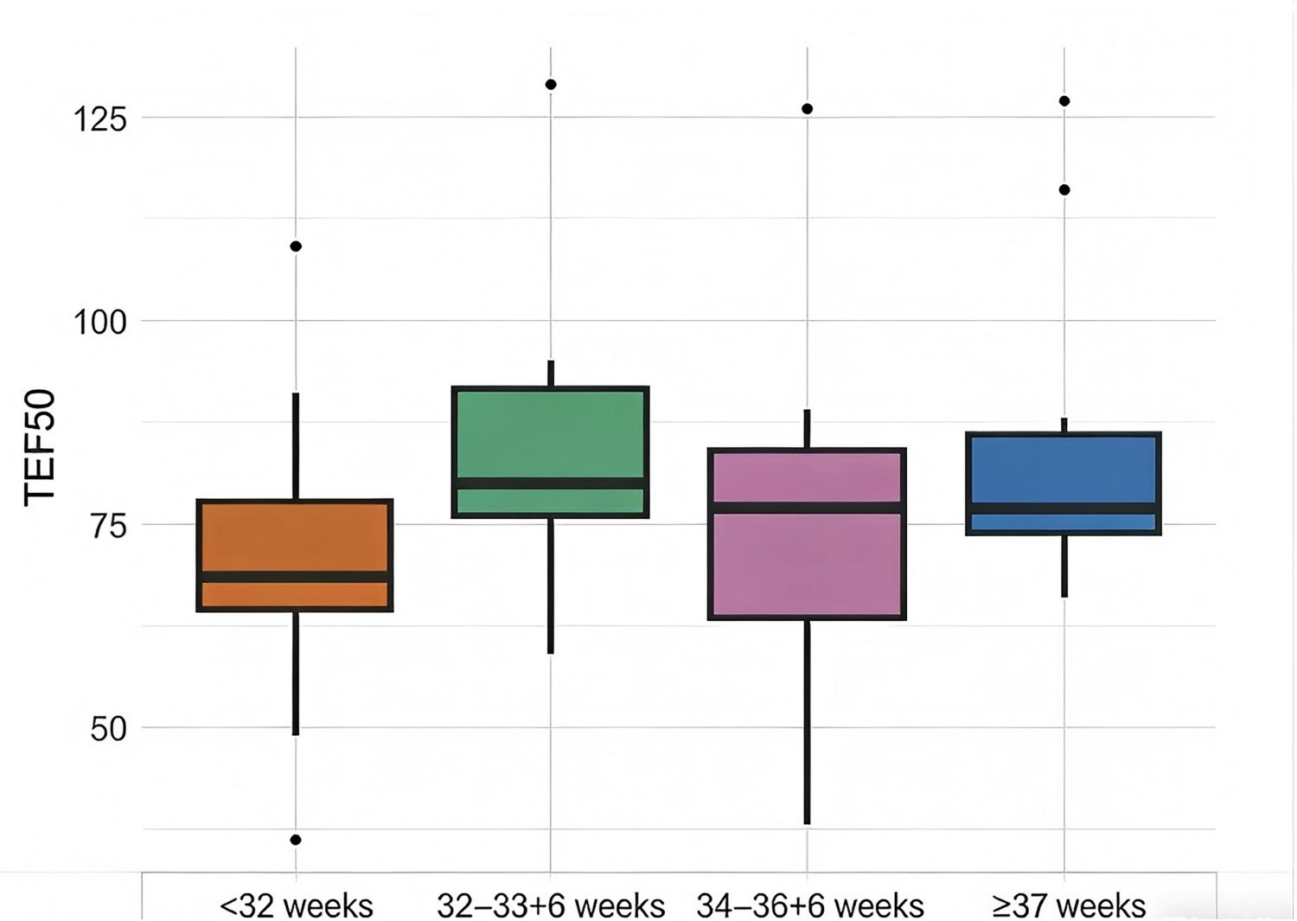
Box-and-whisker plot showing the distribution of expiratory flow at 50% tidal volume (TEF50, mL/s) at 6 months of age among newborns grouped by gestational age quartiles. [Vertical axis: TEF50 values, units mL/s; median value in the < 32 weeks group (84.2 mL/s) was lower than that in the ≥ 37 weeks group (75.1 mL/s), *P* < 0.05].

Line Chart: VT showed an increasing trend with age across different gestational age groups. The steepest slope was observed in the < 32-week group (Figure 1). RR exhibited a decreasing trend with age, and the steepest decline was in the 32–33 + 6-week group. TEF50 demonstrated an increasing trend with age. At 6 months, it remained lower in the < 32-week group compared to the ≥ 37-week group (Figure 2).Boxplot: At baseline, the distributions of VT and TPEF/TE were significantly lower in the < 32-week group compared to the ≥ 37-week group, while RR was significantly higher (Figure 3). By 6 months of age, the differences in pulmonary function distributions across groups narrowed. Only TEF50 remained lower in the < 32-week group (Figure 4).

## 4 Discussion

### 4.1 Physiological mechanisms and clinical significance of baseline pulmonary function differences

This study confirms that at baseline, preterm infants exhibit gestational age-dependent pulmonary function deficits, manifested as reduced tidal volume (VT), decreased ratio of time to peak expiratory flow to total expiratory time (TPEF/TE), and elevated respiratory rate (RR). These findings are consistent with decreased ventilatory efficiency due to immature alveolar differentiation and insufficient pulmonary surfactant (16–19). Among these, extremely preterm infants (< 32 weeks) exhibited significantly lower TPEF/TE, suggesting elevated early small airway resistance and providing pathophysiological evidence for their increased long-term asthma risk (20, 21). Conversely, the 32–33 + 6 weeks group exhibited the highest RR, potentially reflecting “transitional immaturity” in lung development at this gestational age, where alveolar proliferation remains incomplete and respiratory center regulation is still underdeveloped, necessitating an increased respiratory rate to maintain gas exchange. This finding fills a gap in previous research that focused solely on extremely preterm infants.

### 4.2 Core patterns and influencing factors of longitudinal compensatory recovery

This study is the first to validate through dual-group analysis the compensatory pattern that “the younger the gestational age, the faster the rate of pulmonary function recovery”: The VT growth rate in the < 32-week group (0.23 mL/month) was 2.5 times that of term infants, potentially reflecting stronger lung tissue proliferation potential in extremely preterm infants and the maturation-promoting effects of early interventions (e.g., noninvasive ventilation, surfactant administration) (22–27). However, caution is warranted: although most indicators catch up to those of term infants by 6 months of age, the maximal expiratory flow at 50% of the forced vital capacity (TEF50) in the < 32-week group remains significantly lower. TEF50 is a core indicator of small airway function. This persistent lag suggests ongoing structural remodeling of the small airways in extremely preterm infants, consistent with previous findings that “small airway abnormalities are initiating factors for long-term chronic respiratory diseases” (28, 29), indicating the need for targeted monitoring of this population.

Analysis of perinatal factors revealed a positive correlation between birth weight and VT growth, consistent with the mechanism that “intrauterine nutritional reserves influence lung tissue development” (30). Maternal gestational diabetes accelerated VT growth, potentially linked to fetal intrauterine hyperglycemia stimulating pulmonary surfactant synthesis (31–34), though balanced against long-term metabolic disorder risks (35). This finding offers new perspectives for lung function management in preterm infants born to diabetic mothers.

Contrary to the common expectation of improvement with age, TPEF/TE declined from 0 to 6 months in the 32–33 + 6 weeks, 34–36 + 6 weeks, and term groups. This trend reflects normal postnatal maturation of breathing patterns. At term-equivalent age, infants show rapid, shallow breathing with a high proportion of early expiratory flow, leading to a higher TPEF/TE. With maturation, breathing becomes slower and deeper, and peak expiratory flow shifts toward mid-to-late expiration, causing a physiological reduction in TPEF/TE. The < 32 weeks group had a much smaller decline, indicating persistent immaturity of small airway dynamics in extremely preterm infants.

### 4.3 Research innovation and clinical translation value

The core innovations of this study are threefold: a. A dual-grouping approach was adopted, refining preterm subgroups through quadripartite stratification while enhancing statistical power via tripartite grouping to ensure conclusion stability. b. Focusing on small airway function in extremely preterm infants, it clarified the catch-up delay characteristics of TEF50, addressing the lack of longitudinal follow-up for this indicator in previous studies. c. Inclusion of covariates like maternal gestational diabetes revealed interactions between gestational age and perinatal factors.

For clinical translation, this study proposes individualized follow-up strategies: a. Extremely preterm infants (< 32 weeks): Monitor TEF50 every 3 months with early intervention (e.g., nebulized bronchodilators, pulmonary rehabilitation) as needed. b. Preterm infants at 32–36 + 6 weeks: Focus on monitoring the decline in respiratory rate (RR) before 3 months of age to prevent hyperventilation injury. c. Term infants: Routine follow-up is sufficient.

This strategy reduces the long-term risk of respiratory diseases in preterm infants and improves their quality of life.

Our findings of gestational age-dependent baseline deficits in VT, TPEF/TE, and RR are consistent with previous cross-sectional studies in preterm cohorts (36). The faster VT catch-up in extremely preterm infants supports the concept of accelerated compensatory lung growth after preterm birth (37). Importantly, the persistent reduction in TEF50 at 6 months in infants < 32 weeks extends prior short-term observations and confirms ongoing small airway impairment, consistent with reports linking extreme prematurity to increased risk of recurrent wheezing and asthma in later childhood (38, 39).

### 4.4 Limitations and future directions

This study has limitations including a small sample size (especially 19 subjects in the < 32 weeks group), a single-center design, and exclusion of factors such as feeding methods and environmental pollutant exposure. Future studies should include multicenter, large-sample designs with extended follow-up to 1–2 years to analyze the association between lung function and long-term respiratory diseases. Additionally, integrating technologies such as lung ultrasound and genetic testing may help identify early predictive markers for small airway dysfunction.

## 5 Conclusions

Significant differences exist in baseline pulmonary function between preterm infants of varying gestational ages and term infants. Preterm infants exhibit gestational age-dependent compensatory recovery, with faster recovery rates at earlier gestational ages. However, small airway function (TEF50) remains delayed at 6 months in extremely preterm infants born < 32 weeks. Gestational age, birth weight, and maternal gestational diabetes are key influencing factors. Clinicians should develop individualized follow-up strategies based on gestational age, with a focus on enhancing monitoring and intervention for small airway function in extremely preterm infants to improve long-term outcomes.

## Data Availability

The original contributions presented in the study are included in the article/Supplementary Material, further inquiries can be directed to the corresponding author.
